# Status of COVID-19 vaccination in patients undergoing dialysis in China: a national cross-sectional study (2022)

**DOI:** 10.3389/fpubh.2025.1478745

**Published:** 2025-07-17

**Authors:** Ping Li, Delong Zhao, Hong Wang, Yue Niu, Jianhui Zhou, Weicen Liu, Xueying Cao, Zhifang Ma, Sai Pan, Yuan Liang, Xuefeng Sun, Guangyan Cai, Xiangmei Chen

**Affiliations:** Department of Nephrology, First Medical Center of Chinese PLA General Hospital, State Key Laboratory of Kidney Diseases, National Clinical Research Center for Kidney Diseases, Beijing Key Laboratory of Medical Devices and Integrated Traditional Chinese and Western Drug Development for Severe Kidney Diseases, Beijing Key Laboratory of Digital Intelligent TCM for the Prevention and Treatment of Pan-Vascular Diseases, Key Disciplines of National Administration of Traditional Chinese Medicine (zyyzdxk-2023310), Beijing, China

**Keywords:** COVID-19, vaccine, dialysis, health policy, cross-sectional study

## Abstract

**Introduction:**

Due to their comorbidities and frequent exposure to healthcare settings, patients undergoing dialysis are at a high risk of developing severe COVID - 19. However, there are no customized vaccination guidelines for this group in China. This study had two aims: to systematically evaluate the current status of COVID - 19 vaccination among Chinese dialysis patients and to offer a basis for policy - making and further research.

**Methods:**

This study was conducted across all provinces in mainland China using the stratified randomization method. Electronic questionnaires were distributed to patients undergoing dialysis.

**Results:**

Conducted as a national cross - sectional study from May to July 2022, it involved 131,149 dialysis patients from 2,865 centers. The study examined vaccination coverage, the barriers to vaccination, and the safety of vaccines. Only 21.0% received ≥1 vaccine dose, predominantly inactivated vaccines (84.5%). Adverse reactions occurred in 19.0%, with higher rates for adenovirus vector vaccines (27.3%) than for recombinant protein (19.4%) and inactivated vaccines (18.5%, *P* < 0.001). Among unvaccinated patients, 53.5% faced institutional barriers (e.g., site refusal or lack of recommendations), while 88.7% had no contraindications. Older age (OR = 1.32, 95% CI 1.28–1.36), female gender (OR = 1.18, 1.14–1.22), and hemodialysis (OR = 1.12, 1.06–1.19) predicted non-vaccination.

**Conclusion:**

In general, this study highlights critical barriers to COVID-19 vaccination in dialysis patients: guideline gaps, patient hesitation, and non-specific vaccination settings. Recommendations include updating guidelines to prioritize this population, training non-specialized staff, and launching dialysis center-based vaccination programs. Future research should investigate vaccine immunogenicity in dialysis patients to refine booster strategies.

## Introduction

Patients undergoing dialysis are at high risk of SARS-CoV-2 infection due to comorbidities and frequent exposed in dialysis centers. A region wide registry study of 3,800 dialysis patients in Belgium reported an 8.9% infection rate and 27% mortality rate (1). Similarly, a US national dialysis provider study found a 5.5% infection rate and 24.9% mortality rate within 15 weeks (2). Meta-analyses further confirmed that dialysis patients have a 10.26-fold higher mortality risk from COVID-19 compared to non-dialysis populations (3). Other studies also shown that dialysis patients have a high infection rate of SARS-CoV-2 (4, 5) and a high mortality rate (4, 6–8). To mitigate this risk, countries such as the United States and Canada prioritized dialysis patients in their vaccination rollout plans (9, 10). By June 2021, 64.5% of US dialysis patients had received at least one vaccine dose (11).

Although some studies have shown that patients undergoing dialysis have an impaired immunologic response to vaccines (12), and the serum antibody response rate and level after COVID-19 vaccination are lower than those of the general population (13–15), vaccination still effectively reduces the risk of SARS-CoV-2 infection and mortality in patients undergoing dialysis (16–18).

*The COVID-19 Pneumonia Prevention and Control Program (ninth edition)* (19) clearly states that the vaccine can reduce COVID-19 infection and disease and is an effective measure to reduce the incidence of illness and death. However, vaccine instruction for some of those vaccines list patients with “severe chronic diseases” as contraindications (20), and the *Technical Guidelines for COVID-19 Vaccination (first edition)* (21) lacks specific guidance for patients undergoing dialysis.

In addition, the vaccination rate and common adverse reactions of patients undergoing dialysis in China are unknown. Vaccination conditions and adverse reactions may vary for patients undergoing dialyses in different regions and with different disease characteristics.

This study aimed to systematically assess the current COVID-19 vaccination status among Chinese dialysis patients through a nationwide cross-sectional survey, thereby addressing a critical gap in domestic data. The primary objective was to quantify vaccination coverage, vaccine types administered, and associated adverse reactions in this high-risk population. Furthermore, the findings were intended to promote national authorities to refine vaccination policies specifically for dialysis patients, leveraging evidence from this study to prioritize their eligibility and safety. Additionally, the data provided foundational insights for future clinical trials and observational studies, ultimately contributing to evidence-based public health strategies during the evolving pandemic.

## Materials and methods

### Study design and survey administration

We conducted a cross-sectional study by sampling 3–4 cites from each province by the stratified randomization method. All hemodialysis (HD) and peritoneal dialysis (PD) patients from dialysis centers within sampled cities were invited to participate in the study. Electronic questionnaires were distributed and collected via Wenjuanxing,^1^ a widely used online survey platform in China that ensures encrypted data transmission and automated response aggregation.

The sample size was estimated using the formula for cross-sectional studies:

$n=\frac{Z^{2*}p^{*}{\left(1-p\right)}^{*}}{e^{2}}\text{DEFF}$

where Z = 1.96 (95% confidence level), *p* = 0.20 (expected vaccination rate based on pilot data), e = 0.01 (margin of error), DEFF = 1.5 (design effect for stratified sampling). This yielded a minimum required sample of 9,220. To account for potential non-response (20%), we targeted 11,525 participants.

The electronic questionnaires were distributed to patients undergoing dialysis through the National Clinical Research Center for Kidney Disease. Patients or their proxies filled in the information anonymously. COVID-19 vaccination status was determined by self-report. The National Clinical Research Center for Kidney Diseases, which was managed by special personnel, collected data and conducted data quality control. The survey period was from May to July 2022.

### Development and validation of the survey instrument

The questionnaire was developed by the National Clinical Research Center for Kidney Diseases Expert Group. The contents included demographic characteristics, COVID-19 vaccination status, modality, vintage, vascular access type, and weekly treatment frequency. Adverse reaction/event following immunization was defined as untoward medical occurrence which follows immunization and which does not necessarily have a causal relationship with the use of the vaccine, according to the causality assessment of an adverse event following immunization (AEFI): user manual for the revised WHO classification, 2nd ed., 2019 update (22). To validate the questionnaire, we employed a comprehensive approach. First, we calculated the content validity index (CVI) by having a panel of 10 experts in nephrology and survey design rate the relevance of each item in the questionnaire. An item - level CVI (I - CVI) of 0.8 or above and a scale - level CVI (S - CVI) of 0.9 or above were considered acceptable. We conducted a testing by getting survey feedback from 200 of patients at two dialysis centers of Chinese PLA General Hospital, which helped finalize the survey objects and methods.

### Data handling and reporting

All data were entered and stored in a national database and provincial sub centers were in charge of data quality by ensuring the consistency between the collated data and raw data collected from patients. Each regional board clarified any ambiguities or inconsistencies.

### Ethical approval

This study was approval by the Medical Ethics Committee of the Chinese People’s Liberation Army General Hospital (Approval No. S2022-292-01). Due to the anonymous data collection and minimal risk to participants, the requirement for written informed consent was waived. All participants received standardized verbal explanations of the study’s purpose and procedures from trained dialysis center staff. Participation was voluntary, and participants could withdraw at any time without penalty. Privacy was strictly protected throughout the study. All the procedures were followed in accordance with the Declaration of Helsinki.

### Statistical analysis

Descriptive analysis was performed. Continuous variables with normal distribution were reported by mean ± standard deviation or median and inter quartile range if not normally distributed, and categorical variables were described as frequencies and percentages. Comparisons between groups or subgroups were performed using t-test/one-way analysis of variance (ANOVA) or Wilcoxon rank-sum test /Kruskal-Wallis test or Chi-Square test following the statistical guideline (23). Multivariate logistic regression was applied to examine the association between vaccination and variables, which included age, gender, dialysis status, and common comorbidities. For subgroup analyses, age was grouped by < 60 years and ≥ 60 years. The questionnaire was designed such that missing values were not allowed. Of the COVID-19 vaccines approved in China, the completion of primary vaccination was defined as receiving three doses of recombinant protein vaccine (i.e., Zhifei); two doses of an inactivated vaccine (i.e., BBIBP-CorV, CoronaVac); one shot of adenovirus vector vaccine (i.e., Ad5-nCoV). The completion of booster vaccination was defined as receiving a booster dose 6 months after the completion of primary immunization. Two sided *p* value <0.05 was considered statistically significant. All analyses were conducted using R Version 4.2.0 (R Foundation, Vienna, Austria).

## Results

### Characteristics of patients

From May to July 2022, the survey was conducted in 2,865 dialysis centers in 104 cities. Questionnaires were sent to 151,168 patients undergoing dialysis and 131,149 valid questionnaires were returned, with a response rate of 86.7%. Based on data from Chinese national renal registry data (CNRDS), there were 877,470 patients undergoing dialysis in China at the end of 2021, which reflect that the sampled data covered roughly 14.9% of the whole population.

The majority patients were 117,747 (89.8%) HD patients and the rest (10.2%) were PD patients. Among them, 79,042 patients were male (60.3%), and the proportion of male patients in HD patients was higher than that in PD patients (61.1% vs. 52.8%, *p* < 0.001). The mean age of patients undergoing dialysis was 54.4 ± 15.4 years, 83,483 patients (63.6%) were younger than 60 years, 12,743 patients (9.7%) were 60–65 years, and 34,923 patients (26.7%) were over 65 years old. The median dialysis vintage was 39 months, and duration of dialysis in HD patients was lower than that in PD patients (39 [16, 78] vs. 45 [16, 95] months, *p* < 0.001). The top three reported causes of ESRD were hypertensive nephropathy (24.1%), primary glomerulonephritis (23.3%), and diabetic nephropathy (22%). The main comorbidities were anemia (44.2%), diabetes (26.7%) and nephrotic syndrome (22.5%; Table 1).

**Table 1:** Characteristics of patients undergoing dialysis.

Characteristic	Overall *n* = 131,149	HD patients *n* = 117,747	PD patients *n* = 13,402
Male	79,042 (60.3%)	71,964(61.1%)	7,078 (52.8%)
Age, mean (SD), y	54.4(15.4)	54.6(15.2)	52.2 (16.9)
Age groups			
<60 y	83,483 (63.7)	74,313 (63.1%)	9,170 (68.4%)
≥60 and <65 y	12,743 (9.7%)	11,689 (9.9%)	1,054 (7.9%)
≥65 y	34,923 (26.6%)	31,745 (27.0%)	3,178 (23.7%)
Dialysis duration, median [IQR], months	39 [16, 80]	39 [16, 78]	45 [16, 95]
Causes of dialysis			
Primary glomerular diseases	30,536 (23.3%)	26,687 (22.7%)	3,849 (28.7%)
Diabetic nephropathy	28,824 (22.0%)	26,841 (22.8%)	1,983 (14.8%)
Hypertensive nephropathy	31,672 (24.1%)	28,315 (24.0%)	3,357 (25.0%)
Polycystic kidney	5,482 (4.2%)	5,181 (4.4%)	301 (2.2%)
Systemic lupus erythematosus	1,293 (1.0%)	1,141 (1.0%)	152 (1.1%)
Henoch-Schonlein purpuric nephritis	874 (0.7%)	759 (0.6%)	115 (0.9%)
Obstructive nephropathy	975 (0.7%)	880 (0.7%)	95 (0.7%)
Drug-induced renal impairment	3,094 (2.4%)	2,818 (2.4%)	276 (2.1%)
Interstitial nephritis	613 (0.5%)	532 (0.5%)	81 (0.6%)
Pyelonephritis	2,435 (1.9%)	2,213 (1.9%)	222 (1.7%)
ANCA-Associated Vasculitis Renal Injury	593 (0.5%)	524 (0.4%)	69 (0.5%)
Anti-GBM disease	275 (0.2%)	241 (0.2%)	34 (0.3%)
Comorbidity			
Diabetes	35,057 (26.7%)	32,446 (27.6%)	2,611 (19.5%)
Myocardial infarction	4,623 (3.5%)	4,237 (3.6%)	386 (2.9%)
Cerebral infarction or hemorrhage	9,444 (7.2%)	8,687 (7.4%)	757 (5.6%)
Chronic obstructive pulmonary disease	1,086 (0.8%)	983 (0.8%)	103 (0.8%)
Pulmonary infection	4,104 (3.1%)	3,604 (3.1%)	500 (3.7%)
Anemia	58,009 (44.2%)	50,294 (42.7%)	7,715 (57.6%)
CKD-MBD	19,697 (15.0%)	17,692 (15.0%)	2,005 (15.0%)
Fracture	3,462 (2.6%)	3,188 (2.7%)	274 (2.0%)
Malignancy	1,751 (1.3%)	1,590 (1.4%)	161 (1.2%)
Autoimmune diseases	2,309 (1.8%)	2,033 (1.7%)	276 (2.1%)
Previous COVID-19 infection	98 (0.1%)	95 (0.1%)	3 (0.02%)

HD, Hemodialysis. PD, Peritoneal dialysis. CKD-MBD, Chronic Kidney Disease - Mineral and Bone Metabolism Disorder.

Among the HD patients, 71.3% of the dialysis access was autogenous arteriovenous fistula, 75.1% of the dialysis frequency was three times per week, 92.9% of the dialysis duration was 4 h, and 92.4% of the dialysis room was large open room. During the epidemic, most of the patients chose either public transportation (44.6%) or private car (42.7%; Supplementary Table 1).

Only a small number of PD patients (7.3%) used automated peritoneal dialysis machines. The main follow-up method for PD patients was hospital follow-up (75.9%), followed by telephone follow-up (59.9%) and online follow-up (40.5%). Most PD patients were followed up for 1–3 months (44.0%) or less than 1 month (38.5%). About half of PD patients (49.6%) visited the outpatient clinic by appointments. More than half of PD patients (52.7%) had no fixed hospitalization time, and they chose to be hospitalized only if needed (Supplementary Table 2).

### COVID-19 vaccination status in patients undergoing dialysis

**Figure 1:**
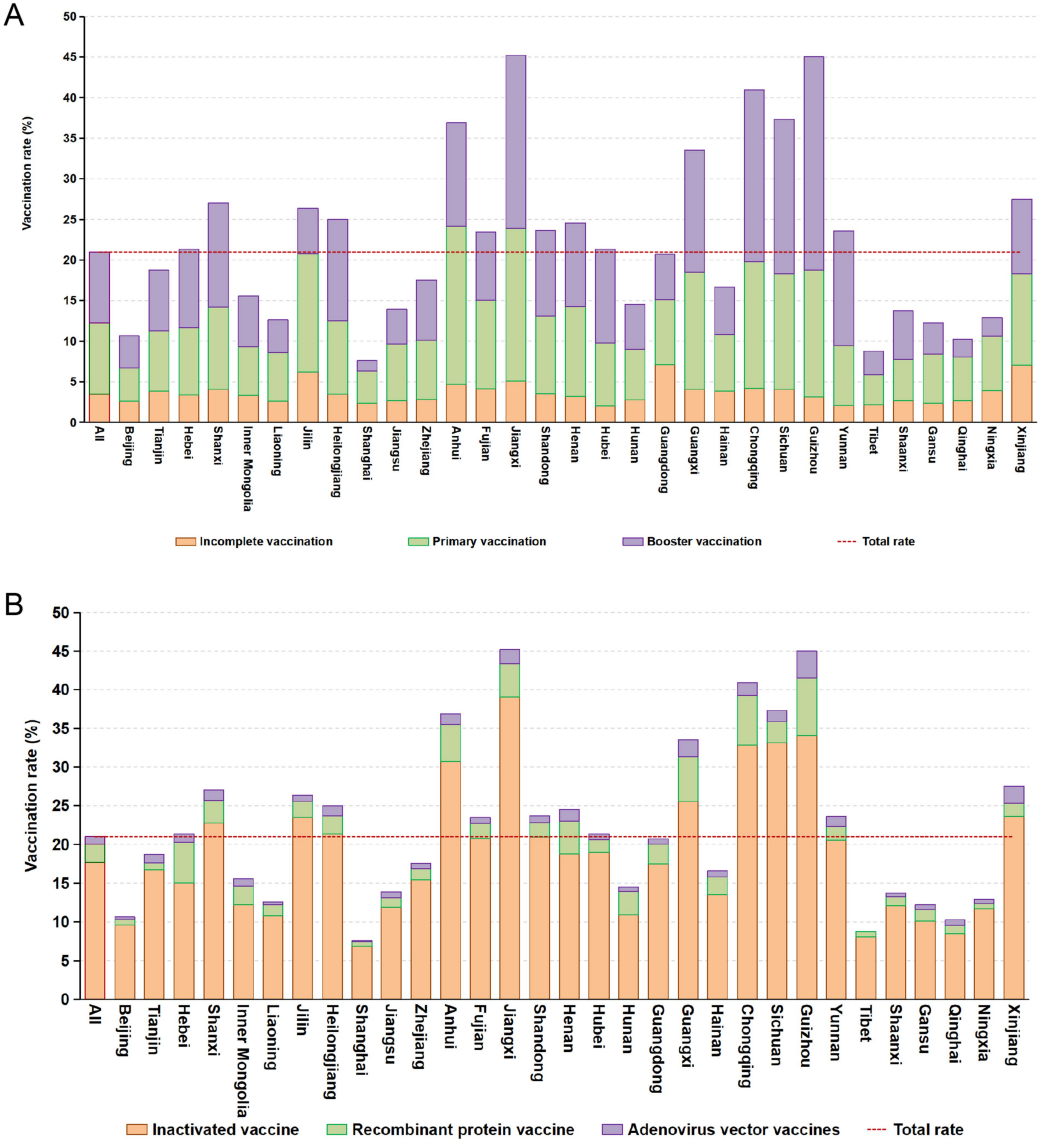
Coverage rates of SARS-CoV-2 vaccines in provinces of China **(A)** and distribution of vaccine types in administrative regions **(B)**.

A total of 27,511 patients undergoing dialysis were vaccinated at least once, and the vaccination rate was 21.0%. Among them, 16.8% of the patients had not completed the primary vaccination, 41.8% of the patients completed the primary vaccination, and 41.5% of the patients completed the booster vaccination. Among the 31 administrative regions, Jiangxi province had the highest vaccination rate, and 16 provinces had vaccination rates higher than the total rate (Figure 1A).

Most vaccinated patients (84.5%) received the inactivated vaccine, 11.1% received the recombinant protein vaccine, and 4.5% received the adenovirus vector vaccine. Among the patients who received the inactivated vaccine, 40.8% completed the primary immunization vaccination and 45.3% completed the booster immunization vaccination. Among the patients who received the recombinant protein vaccine, 45.9% completed the primary immunization vaccination and 16.1% completed the booster immunization vaccination. Among the patients vaccinated with the adenovirus vector vaccine, 36.2% completed the primary immunization vaccination and 63.8% completed the booster immunization vaccination. The coverage rates of different types of COVID-19 vaccines by administrative regions of China were shown in Figure 1B.

#### Adverse reaction

**Figure 2:**
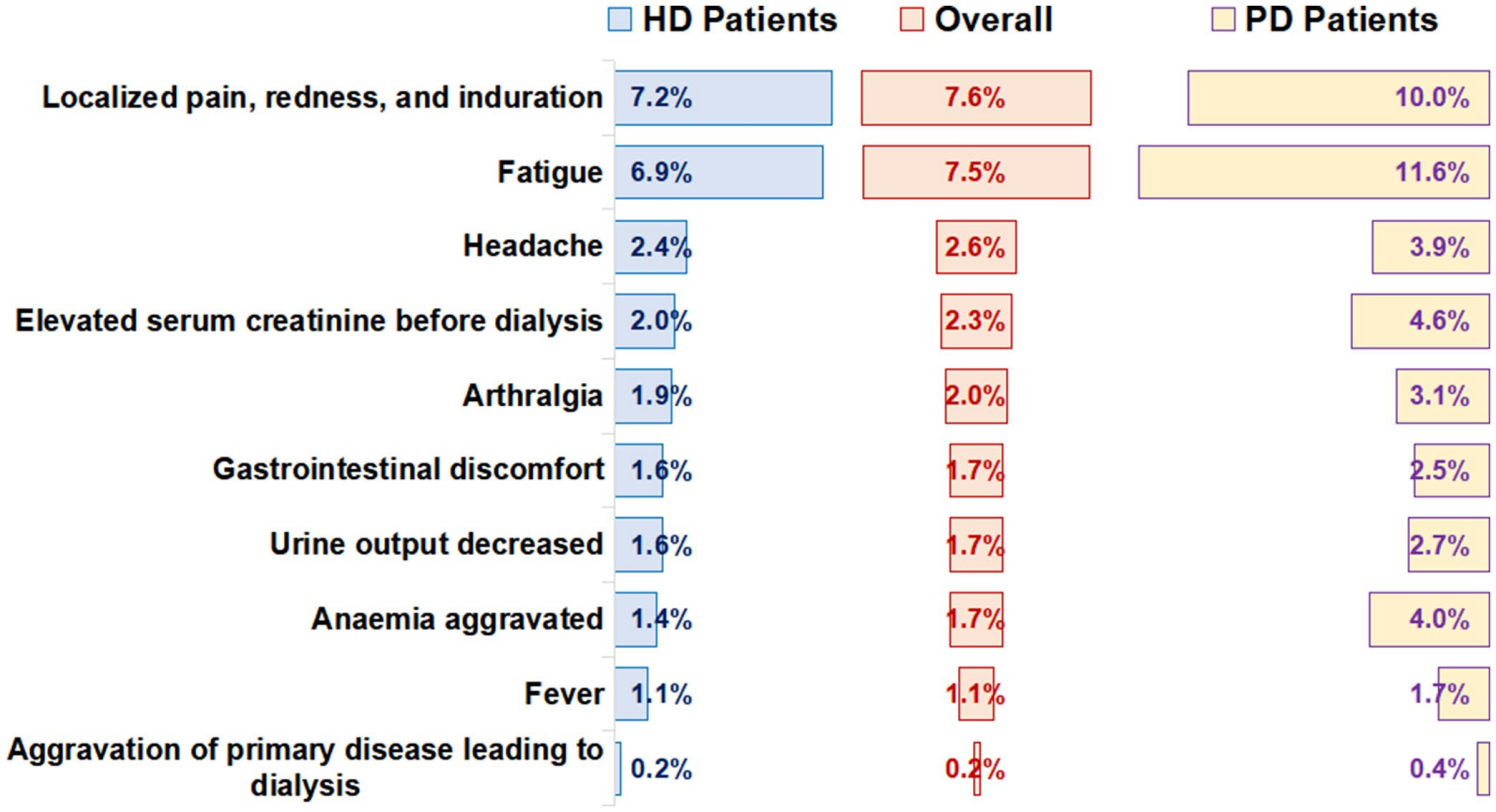
Adverse reactions after receiving the SARS-CoV-2 vaccines.

The number of patients that reported adverse reactions was 5,215 (19.0%). Among them, the most common local adverse reactions were localized pain, redness, and induration (7.6%), and the most common systemic adverse reaction was fatigue (7.5%; Figure 2). The incidence of total adverse reactions in PD patients was higher than that in HD patients (27.5% vs. 17.9%, *p* < 0.001). In addition, we found that the incidence of adverse reactions was lower in male patients and older adult patients (Supplementary Tables 3, 4). Incidence of adverse reactions in different vaccination completion status was shown in Supplementary Table 5.

There were differences in adverse reactions among different types of vaccines. The incidence of adverse reactions of the adenovirus vector vaccine (27.3%) was higher than that of the recombinant protein vaccine (19.4%) and inactivated vaccine (18.5%, *p* < 0.001; Table 2).

**Table 2:** Incidence of adverse reactions of different types of COVID-19 vaccines in patients undergoing dialysis.

	Inactivated vaccines *n* = 23,232	Recombinant protein vaccines *n* = 3,046	Adenovirus vector vaccines *n* = 1,233	*p*
Total adverse reactions	4,287 (18.5%)	592 (19.4%)	336 (27.3%)	<0.001
Localized pain, redness, and induration	1773 (7.6%)	205 (6.7%)	100 (8.1%)	0.16
Fatigue	1,705 (7.3%)	217 (7.1%)	128 (10.4%)	<0.001
Headache	592 (2.5%)	72 (2.4%)	52 (4.2%)	0.001
Elevated serum creatinine before dialysis	481 (2.1%)	88 (2.9%)	62 (5.0%)	<0.001
Arthralgia	456 (2.0%)	64 (2.1%)	37 (3.0%)	0.04
Gastrointestinal discomfort	394 (1.7%)	47 (1.5%)	29 (2.4%)	0.17
Urine output decreased	337 (1.5%)	83 (2.7%)	49 (4.0%)	<0.001
Anemia aggravated	385 (1.7%)	52 (1.7%)	34 (2.8%)	0.02
Fever	251 (1.1%)	32 (1.1%)	30 (2.4%)	<0.001
Hematuria or tea-colored, soy sauce colored urine	52 (0.2%)	9 (0.3%)	7 (0.6%)	0.051

In the hemodialysis subgroup (*p* < 0.001), gender subgroup (*p* < 0.001) and age subgroup (*p* < 0.001 in patients aged 60 years and older, *p* = 0.04 in patients under 60 years of age), the incidence of adverse reactions of patients receiving the adenovirus vector vaccine was higher compared to the other two vaccines (Supplementary Table 6).

### Reasons for non-vaccination in patients undergoing dialysis

For patients who did not receive a COVID-19 vaccine, the main reason was “patients or their family members did not receive the vaccine for fear of affecting their health” (35.2%), followed by “the vaccine injection site refused vaccination because the patient was on dialysis” (29.2%) and “Health care providers did not advise patients to get vaccinated” (24.3%). Only 4.4% of patients did not receive vaccination due to contraindications (Supplementary Figure 1). Among unvaccinated patients, 88.7% had no contraindications to vaccination, as shown in Supplementary Figure 2. The most common contraindications were thrombocytopenia or bleeding disorders (6.9%), history of neurological reactions to vaccines (4.4%), and active malignancy (e.g., lymphoma/leukemia, 3.5%). Only 0.4% of patients cited pregnancy or immunosuppressive therapy as reasons for non-vaccination (Supplementary Figure 2).

**Figure 3:**
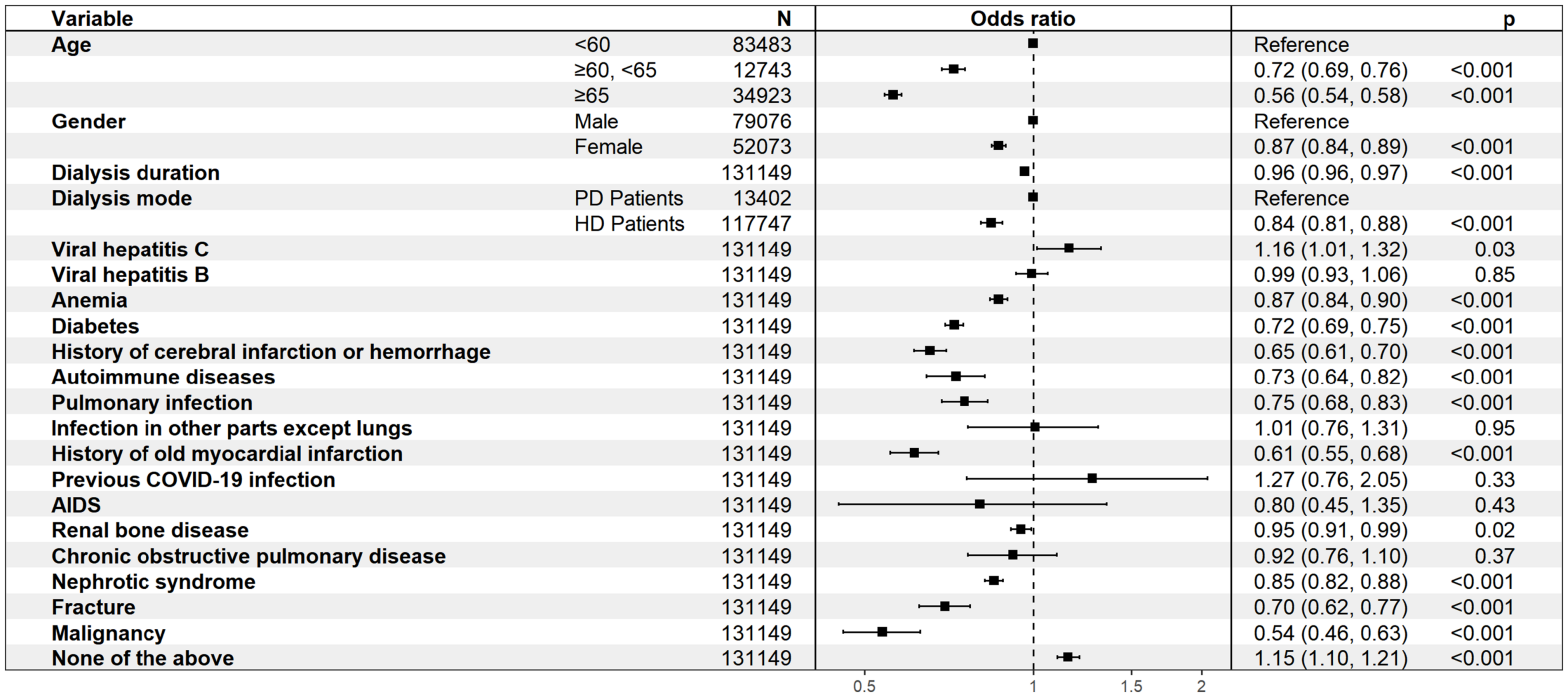
Multivariate analysis of the associations between the included variables and COVID-19 vaccination.

In addition, older age, female gender, and hemodialysis were associated with non-vaccination, and patients without comorbidities were more willing to be vaccinated (Figure 3).

## Discussion

This study was the first large-scale nationwide survey on the COVID-19 vaccination status of patients undergoing dialysis in China. The primary objective was to quantify vaccination coverage, vaccine types administered, and associated adverse reactions in this high-risk population. Furthermore, the findings were intended to promote national authorities to refine vaccination policies specifically for dialysis patients, leveraging evidence from this study to prioritize their eligibility and safety. Additionally, the data provided foundational insights for future clinical trials and observational studies, ultimately contributing to evidence-based public health strategies during the evolving pandemic.

The results of this study show that the COVID-19 vaccination rate among patients undergoing dialysis was 21.0%, which was lower than the rate reported in the United States. The US government announced the availability of COVID-19 vaccination at dialysis clinics on March 25, 2021, and two national dialysis organizations, DaVita In and Fresenius Medical Care, partnered with the Centers for Disease Control and Prevention (CDC) to provide COVID-19 vaccination at their clinics and coordinated the distribution of vaccines to other dialysis organizations. By 13 June 2021, 64.5% of the 483,602 patients undergoing dialysis surveyed had received at least 1 dose of COVID-19 vaccine (11).

The low vaccination rate may be detrimental to patients undergoing dialysis. Due to age and comorbidities, patients undergoing dialysis have a high infection rate and a high mortality rate due to SARS-CoV2 infection. Among the 671 patients undergoing hemodialysis in 6 dialysis centers in the United Kingdom, the rate of SARS-CoV2 infection was 8.9%, and the mortality rate was 27% (24). The SARS-CoV2 infection rate in 7948 routine patients undergoing dialysis in the United States was 5.5% within 15 weeks, and the mortality rate was 24.9% (2). During the COVID-19 epidemic in Wuhan, China, the prevalence of COVID-19 in patients undergoing hemodialysis was 2.15%, which was significantly higher than that of the general population in Wuhan (about 0.5% until March 10, 2020) (25). In a meta-analysis of 348 studies and 382,407 COVID-19 patients with Chronic Kidney Disease (CKD) out of 1,139,979 patients with CKD, patients undergoing dialysis had a significantly higher rate of SARS-CoV-2 infection than non-dialysis CKD patients (105 vs. 16/10,000 person-weeks), and the mortality rate of CKD patients with SARS-CoV-2 infection was 10.26 times that of CKD patients without infection (3). Vaccination reduced the risk of infection and death in patients undergoing dialysis. In a retrospective study in the United States, more than 35,000 patients undergoing hemodialysis had a risk ratio of 0.22 and 0.27 for SARS-CoV-2 after receiving BNT161b2 and mRNA-1,273 vaccines, respectively (16). Modeling of hospitalization data from 3,620 patients undergoing dialysis and 457,160 general population infected with SARS-CoV-2 in France shows that vaccination may have a protective effect against severe COVID-19 in patients undergoing hemodialysis (17). A multi-center clinical observational study of the severity of BNT162b2 and AZD1222 vaccines and COVID-19 in a population of about 5,500 patients undergoing hemodialysis in London found that after adjusting for age, comorbidities and time, the risk ratio for admission with two doses of vaccine was 0.25 and the risk ratio for death was 0.12 compared with unvaccinated patients (18). Therefore, early and rapid vaccination is an absolute priority for the high-risk group of patients undergoing dialysis.

Three interrelated factors emerged as critical drivers of low vaccination uptake: (1) Clinician apprehensions. Due to the inherent nature of their illnesses, dialysis patients have a weakened response to vaccines to varying degrees, which slightly reduces the vaccine’s effectiveness. Meanwhile, the risk of adverse reactions may increase. As a result, healthcare workers question the benefits of vaccinating dialysis patients. (2) Structural Barriers in Vaccine Delivery. Notably, 29.2% of these refusals occurred at non-specialized facilities (e.g., community clinics), where staff often lacked training in managing complex chronic disease patients. Similar challenges were observed in early US nursing home vaccine rollouts, highlighting a global need for targeted provider education. (3) Lack of guideline support. At present, international mRNA vaccines are eligible for those with chronical diseases, and the US CDC considers the contraindications to the COVID-19 vaccine as allergies after receiving a dose of COVID-19 vaccine and previous allergy to vaccine components, otherwise recommends that patients undergoing dialysis and medical personnel be vaccinated with COVID-19 vaccine (9). The recommendations of the Advisory Committee on Immunization Practices in the United States on the allocation of COVID-19 vaccines—the order of vaccination in December 2020 is divided into four groups: 1a, 1b, 1c, and 2, with the older adult, healthcare workers, and patients with high-risk diseases being prioritized (26). The Canadian province of Ontario also ranks patients undergoing dialysis as the second highest priority (10). Part of domestic inactivated COVID-19 vaccines list serious chronic diseases as contraindications, so it’s unclear whether patients undergoing dialysis should be vaccinated, and the national “SARS-CoV-2 Vaccination Technical Guidelines (First Edition)” also lacks specific guidance for dialysis treatment patients (21). The first version of the guidelines has been in effect for years, and the situation of epidemic prevention and control is constantly changing, so it is necessary for the relevant departments to make more detailed and practical recommendations specifically for patients undergoing dialysis, to increase the vaccination rate among patients undergoing dialysis and reduce the risk of infection and death.

In this survey, all patients were vaccinated with vaccines made in China, of which the most vaccinated were inactivated vaccines produced by Sinopharm or Sinovac, accounting for 84.5%, followed by recombinant protein vaccines produced by Zhifei Biologics, accounting for 11.1%, and adenovirus vector vaccines produced by CanSino accounted for the lowest proportion of 4.5%. The incidence of adverse reactions after vaccination was 19.0%, the most common local adverse reactions were local pain, redness and induration, accounting for 7.6%, and the most common systemic adverse reactions were fatigue, accounting for 7.5%. The results were similar to what reported by other countries using same vaccine products. The results of the phase 3 clinical trial of 6,646 cases of Sinovac inactivated vaccine in Turkey aged 18–59 years showed that the incidence of adverse events was 18.9%, the most common systemic adverse event was fatigue (8.2%), and the most common local adverse event was injection site pain (2.4%) (27). Obviously, patients undergoing dialysis did not have serious adverse reactions after vaccination with Chinese-made vaccines, indicating that patients undergoing dialysis were safe and well tolerated after vaccination.

In this study, patients who completed primary immunization or booster immunization were lower, accounting for only 17.5%. In a study of 50 uninfected and 15 HD patients with a history of infection, the immune response rate of 2 doses of vaccine was 89%, the immune response rate after 3 doses of vaccine was 93%, and the immune response rate of 3 doses of vaccine in uninfected people was similar to that of previous infection and 2 doses of vaccine, and the third dose enhanced the response rate of almost all patients, especially those with low antibody titers or partial non-response after 2 doses of vaccine, who had a strong immune response after receiving the 3rd dose (28). A review of 22 studies on early immune responses to COVID-19 vaccination in patients undergoing hemodialysis found that antibody production was observed in 18 to 53% of patients with 1 dose of mRNA vaccine and in 70 to 96% of patients after 2 doses of mRNA vaccine (29). In a meta-analysis of 32 studies of 4,111 patients undergoing dialysis, the total antibody response rate of patients undergoing dialysis was 86%, compared with patients who are not on dialysis, the antibody response rate after the first vaccination was reduced by 39%, the antibody response rate after the second vaccination was reduced by 12%, the low response rate after the second vaccination was significantly lower than the low response rate after the first vaccination (*p* = 0.01), and the subgroup analysis found that the response rate of patients without complete vaccination was 41%, the response rate of patients with complete vaccination was 89%, and the response rate of patients with intensive vaccination was 94%, There was no significant difference between patients on hemodialysis and peritoneal dialysis (87% versus 94%, *p* = 0.2) (30). The response rate of patients undergoing dialysis is positively related to the number of vaccinations, and complete or booster vaccination ought to be highly recommended among patients undergoing dialysis.

In addition, it is of further concern that the response rate is not fully representative of antibody levels. Seroprotection only describes the proportion of patients who exceed the limit of antibody detection but does not provide information on seroconversion (13). A prospective multicenter study found that humoral immune responses were incomplete, delayed, and weakened cellular immune responses in patients undergoing hemodialysis compared with healthy volunteers (14, 15). Several other studies have also found that although most patients undergoing hemodialysis develop significant humoral reactions after vaccination, they are significantly lower than healthy controls. Possible causes are impaired immune function due to primary kidney disease and long-term dialysis treatment. A study of fluid and B-cell responses in patients undergoing hemodialysis found that significantly impaired anti-BNT162b2 responses, delayed (3–4 weeks after strengthening) and reduced anti-S1 IgG and IgA positive responses were found in patients undergoing hemodialysis, compared with healthy controls, 70.5 and 68.2%, respectively (12). The above results and data were derived from mRNA vaccine-related studies. However, due to the limited availability of immunogenicity testing (e.g., antibody titers) in the study population, the immunogenicity of inactivated vaccines was not investigated. Therefore, the current vaccination regimen for patients undergoing dialysis might not be optimal and would urgently need review and possible improvement.

This analysis is subject to at least three limitations. First, this study was conducted in patients undergoing dialysis and did not include any other patients who are not on dialysis for comparison. Second, the proportion of patients with detected antibody titers was low. Finally, the status of vaccination was self-reported by patients, and we did not validate the accuracy of dose information.

This study was initiated and organized by the National Clinical Research Center for Kidney Diseases. The survey was distributed and collected covers 31 administrative regions in mainland, China, and the basic characteristics such as gender, age, dialysis mode, and dialysis frequency closely represent to the overall dialysis status in China, and comprehensive, and accurate information may help gain an in-depth understanding of the current situation of hemodialysis treatment during COVID-19 epidemic in China, and provide the basis for the research and making relevant policies, guidelines and consensus.

## Conclusion

This study highlights critical barriers to COVID-19 vaccination in dialysis patients: guideline gaps, patients hesitation, and non-specific vaccination settings. Recommendations include updating guidelines to prioritize this population, training non-specialized staff, and launching dialysis center-based vaccination programs. Future research should investigate vaccine immunogenicity in dialysis patients to refine booster strategies.

## Data Availability

The raw data supporting the conclusions of this article will be made available by the authors, without undue reservation.

## Footnotes

^1^ www.wjx.cn
