# Supplementary material for: Status of COVID-19 vaccination in patients undergoing dialysis in China: a national cross-sectional study (2022)
Source: Front Public Health. 2025 Jul 17;13:1478745. doi: 10.3389/fpubh.2025.1478745 (PMC12310671; doi:10.3389/fpubh.2025.1478745)
Supplement: Supplementary file 1 [file Data_Sheet_1.docx]

**Supplementary Table 1. Treatment and follow-up of HD patients**

|  | **HD Patients  n=117,747** |
| --- | --- |
| **Dialysis access** |  |
| Artificial vascular fistula | 13,287 (11.3%) |
| Autogenous arteriovenous fistula | 83,953 (71.3%) |
| Long-term intravenous catheterization | 16,910 (14.4%) |
| Temporary intravenous catheterization | 2,376 (2.0%) |
| Others | 1,221 (1.0%) |
| **The frequency of dialysis** |  |
| 4 times per week | 911 (0.8%) |
| 3 times per week | 88,383 (75.1%) |
| 5 times every two weeks | 9,183 (7.8%) |
| Twice per week | 17,679 (15.0%) |
| Once per week | 734 (0.6%) |
| Others | 857 (0.7%) |
| **The duration of each dialysis** |  |
| 4 hours | 109,378 (92.9%) |
| 3 hours | 4,081 (3.5%) |
| 2 hours | 248 (0.2%) |
| 1 hour | 22 (0.0%) |
| Other | 4,018 (3.4%) |
| **Dialysis room** |  |
| Single room | 4,135 (3.5%) |
| Screen separation (complete separation by screen or other facility) | 4,825 (4.1%) |
| Large open room (open space or semi-high wall isolation only) | 108,787 (92.4%) |
| **The trip mode to dialysis room** |  |
| Walking | 16,591 (14.1%) |
| Bicycle | 16,119 (13.7%) |
| Private car | 50,323 (42.7%) |
| Public transportation | 52,498 (44.6%) |

HD, Hemodialysis.

**Supplementary Table 2. Treatment and follow-up of PD patients**

|  | **PD Patients  n=13,402** |
| --- | --- |
| **PD modality** |  |
| Manual Fluid Change | 12,421 (92.7%) |
| Machine Fluid Change | 981 (7.3%) |
| **Frequency of follow-up** |  |
| < 1 month | 5,162 (38.5%) |
| 1-3 months | 5,895 (44.0%) |
| About half a year | 1,342 (10.0%) |
| About 1 year | 201 (1.5%) |
| No follow-up | 802 (6.0%) |
| **The follow-up method** |  |
| Telephone follow-up | 8,024 (59.9%) |
| Online follow-up | 5,429 (40.5%) |
| Follow-up in the hospital | 10,172 (75.9%) |
| Follow-up in patient's home | 2,104 (15.7%) |
| **Frequency of outpatient visits** |  |
| < 1 month | 5,522 (41.2%) |
| 1-3 months | 5,855 (43.7%) |
| About half a year | 1,096 (8.2%) |
| About 1 year | 53 (0.4%) |
| No fixed time, and will go to hospital if there is a problem. | 876 (6.5%) |
| **Outpatient appointment system** |  |
| Always make appointments | 6,645 (49.6%) |
| Mostly make appointments | 3,899 (29.1%) |
| Rarely make appointments | 1,068 (8%) |
| No appointment system | 1,789 (13.3%) |
| **Frequency of hospitalization** |  |
| 1 month | 428 (3.2%) |
| 1-3 months | 1,840 (13.7%) |
| About half a year | 3,246 (24.2%) |
| About 1 year | 820 (6.1%) |
| No fixed time, and will go to hospital if there is a problem | 7,069 (52.7%) |

PD, Peritoneal dialysis. RPM, Remote Patient Management.

**Supplementary Table 3. Incidence of adverse reactions stratified by gender.**

|  | **Male**  **n=17,336** | **Female**  **n=10,175** | ***P*** |
| --- | --- | --- | --- |
| Total adverse reactions | 2,897 (17.6%) | 1,974 (20.7%) | <.001 |
| Localized pain, redness, and induration | 1,062 (6.1%) | 938 (9.2%) | <.001 |
| Fatigue | 1,181 (6.8%) | 757 (7.4%) | .053 |
| Headache | 391 (2.3%) | 291 (2.9%) | .002 |
| Elevated serum creatinine before dialysis | 442 (2.5%) | 180 (1.8%) | <.001 |
| Arthralgia | 325 (1.9%) | 221 (2.2%) | .10 |
| Gastrointestinal discomfort | 283 (1.6%) | 196 (1.9%) | .08 |
| Urine output decreased | 313 (1.8%) | 135 (1.3%) | .003 |
| Anaemia aggravated | 267 (1.5%) | 168 (1.7%) | .51 |
| Fever | 211 (1.2%) | 123 (1.2%) | 1 |
| Hematuria or tea-colored, soy sauce colored urine | 49 (0.3%) | 18 (0.2%) | .112 |

**Supplementary Table 4. Incidence of adverse reactions stratified by age group.**

|  | **Under 60**  **n=20,317** | **At or above 60**  **n=7,194** | ***P*** |
| --- | --- | --- | --- |
| Total adverse reactions | 3,957 (20.2%) | 914 (14.3%) | <.001 |
| Localized pain, redness, and induration | 1,638 (8.1%) | 362 (5.0%) | <.001 |
| Fatigue | 1,557 (7.7%) | 381 (5.3%) | <.001 |
| Headache | 564 (2.8%) | 118 (1.6%) | <.001 |
| Elevated serum creatinine before dialysis | 485 (2.4%) | 137 (1.9%) | .02 |
| Arthralgia | 434 (2.1%) | 112 (1.6%) | .003 |
| Gastrointestinal discomfort | 340 (1.7%) | 139 (1.9%) | .17 |
| Urine output decreased | 356 (1.8%) | 92 (1.3%) | .008 |
| Anaemia aggravated | 356 (1.8%) | 79 (1.1%) | <.001 |
| Fever | 265 (1.3%) | 69 (1.0%) | .03 |
| Hematuria or tea-colored, soy sauce colored urine | 57 (0.3%) | 10 (0.1%) | .051 |

**Supplementary Table 5.** **Incidence of adverse reactions** **in different vaccination completion status.**

|  | **Incomplete vaccination**  **n=4,222** | **Primary vaccination**  **n=10,932** | **Booster vaccination**  **n=12,357** |
| --- | --- | --- | --- |
| Total adverse reactions | 941 (23.5%) | 2,035 (19.7%) | 1,895 (16.2%) |
| Localized pain, redness, and induration | 242 (5.7%) | 833 (7.6%) | 925 (7.5%) |
| Fatigue | 395 (9.4%) | 852 (7.8%) | 691 (5.6%) |
| Headache | 176 (4.2%) | 297 (2.7%) | 209 (1.7%) |
| Elevated serum creatinine before dialysis | 158 (3.7%) | 262 (2.4%) | 202 (1.6%) |
| Arthralgia | 113 (2.7%) | 228 (2.1%) | 205 (1.7%) |
| Gastrointestinal discomfort | 133 (3.2%) | 196 (1.8%) | 150 (1.2%) |
| Urine output decreased | 92 (2.2%) | 181 (1.7%) | 175 (1.4%) |
| Anaemia aggravated | 103 (2.4%) | 189 (1.7%) | 143 (1.2%) |
| Fever | 79 (1.9%) | 149 (1.4%) | 106 (0.9%) |
| Hematuria or tea-colored, soy sauce colored urine | 15 (0.4%) | 33 (0.3%) | 19 (0.2%) |

**Supplementary Table 6.** **Incidence of total adverse reactions of different types of COVID-19 vaccines in subgroup**

|  | **Inactivated**  **vaccines** | **Recombinant protein vaccines** | **Adenovirus vector vaccines** | ***P*** |
| --- | --- | --- | --- | --- |
| Patients on hemodialysis | 17.30% | 18.80% | 26.70% | <.001 |
| Patients on peritoneal dialysis | 27.4% | 26.6% | 31.4% | .57 |
| Male patients | 17.2% | 18.8% | 27.6% | <.001 |
| Female patients | 20.5% | 20.6% | 26.7% | .006 |
| Patients aged 60 years and older | 15.0% | 13.9% | 21.7% | .01 |
| Patients under 60 years of age | 19.7% | 21.0% | 28.5% | <.001 |


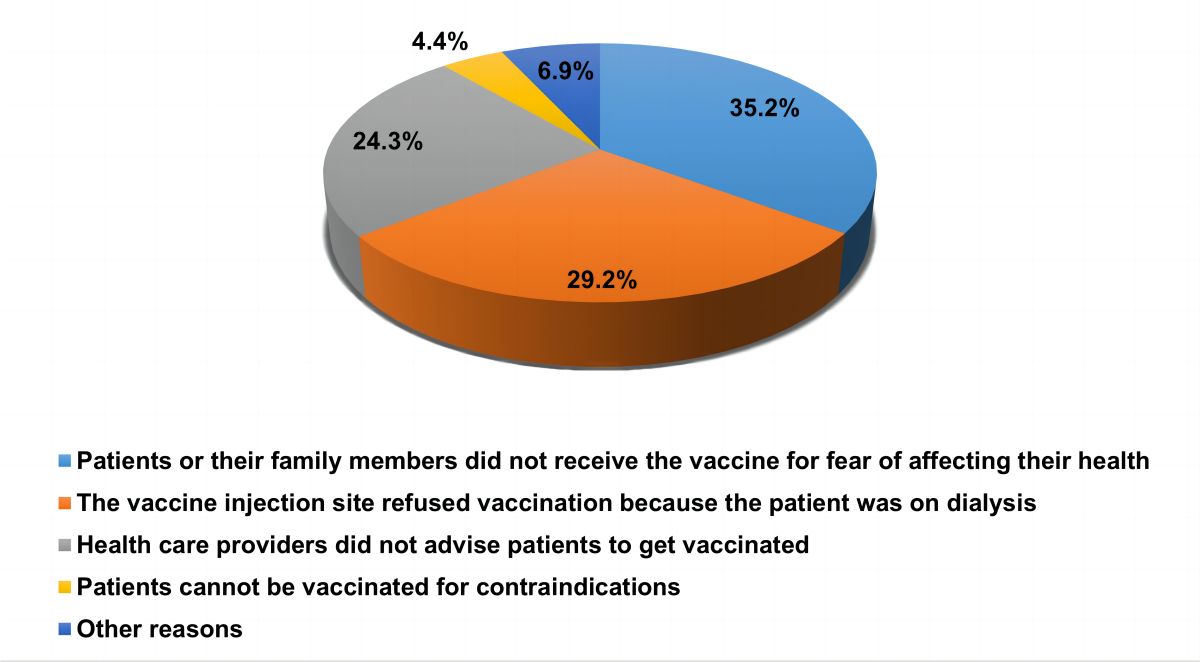


**Supplementary Figure 1. Reasons for non-vaccination in dialysis patients**


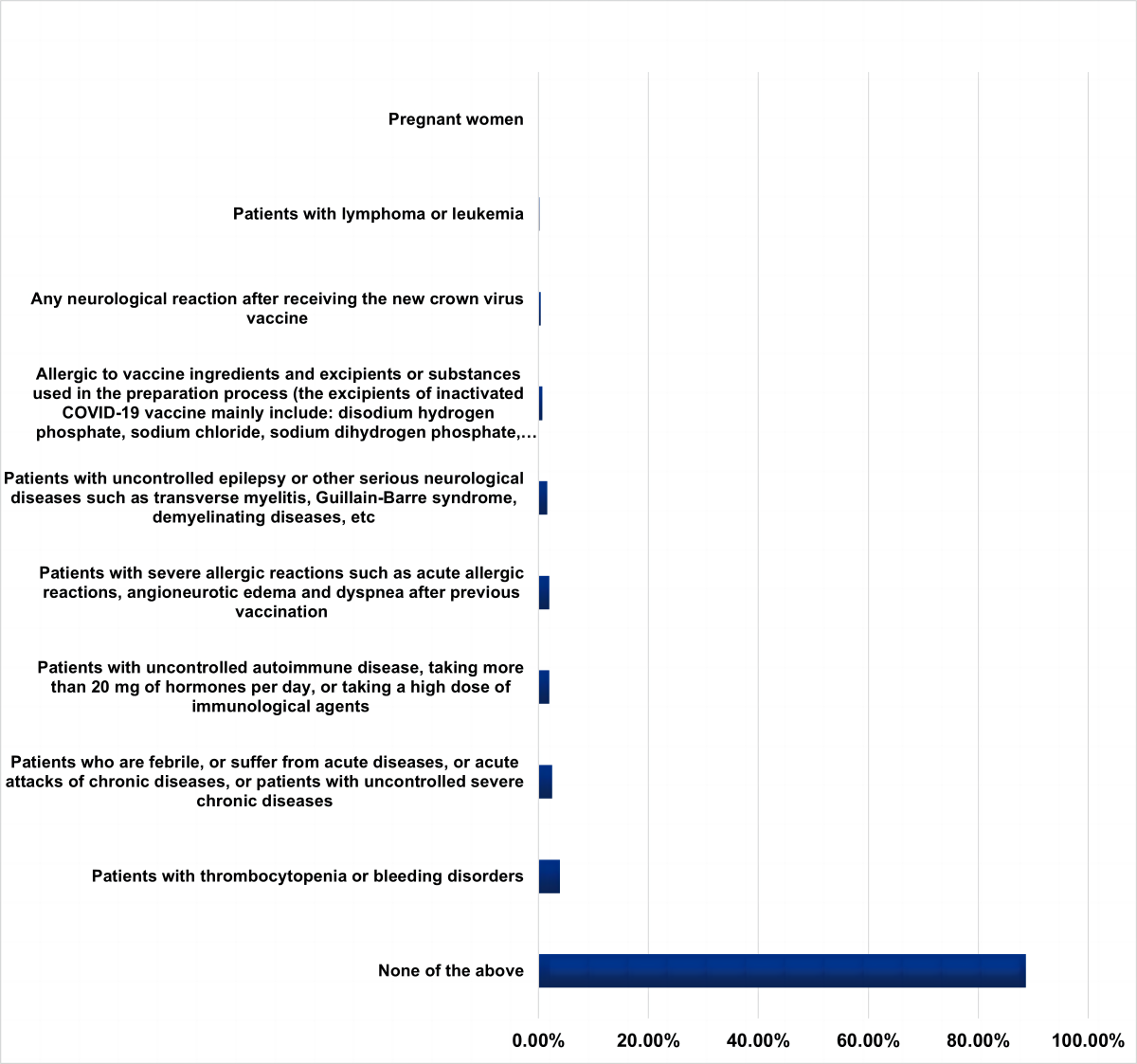


**Supplementary Figure 2. Contraindications to vaccination in unvaccinated patients**
